# Certain immune parameters may have a significant impact on suicidal behaviour - a naturalistic study among psychiatric in-patients

**DOI:** 10.1192/j.eurpsy.2024.721

**Published:** 2024-08-27

**Authors:** V. Voros, E. Saghy, C. Molnar, M. Kovacs, B. Peto, S. Kovacs, A. Zemplenyi, S. Fekete, T. Tenyi, P. Osvath

**Affiliations:** ^1^Department of Psychiatry and Psychotherapy; ^2^Center for Health Technology Assessment and Pharmacoeconomic Research, Faculty of Pharmacy, University of Pecs, Pecs, Hungary

## Abstract

**Introduction:**

Several research already proved the role of certain immunological factors (neutrophil-lymphocyte (NLR), monocyte-lymphocyte (MLR) and platelet-lymphocyte (PLR) ratio, and C-reactive protein (CRP)) in the background of suicidal behaviour.

**Objectives:**

The aim of this research was to study the association between routinely measurable low-grade inflammation parameters and suicidal behaviour among patients in the acute psychiatric care setting.

**Methods:**

The study population included psychiatric in-patients (N=100) consecutively treated with depressive disorders and/or suicidal behaviour in a University Clinic between December 1, 2020 and December 31, 2021. Three different patient-groups were generated based on their suicidal behaviour: suicide attempters (N=55) including *recent attempters*(N=36) and *past attempters* (N=19) and *non-suicidal patients* (N=45), who never had a suicide attempt. Basic socio-demographic data, the severity of depression and immunological parameters (white blood cell count: lymphocytes, monocytes, neutrophil, eosinophil, basophil granulocytes; thrombocytes; C-reactive protein) were recorded.Descriptive analyses and multivariate regression model were performed with RStudio version 4.2.3.

**Results:**

CRP was significantly higher (2.00 vs. 1.00; p=0.007) in suicidal patients (N=55), however other immunological parameters did not differ significantly between the suicidal and the non-suicidal groups (NLR: 2.02 vs. 2.19; MLR: 0.22 vs. 0.11; PLR: 118 vs. 130). NLR and MLR showed significantly higher values (NLR: 2.83 vs. 1.93, p=0.021; MLR: 0.28 vs. 0.11, p=0.01) for those who currently attempted suicide (N=36) compared to the patients with no or past suicide attempt (N=64). In the regression analysis, the NLR and MLR showed significantly higher values in current suicide attempters even when gender, age, suicidal risk and severity of depression were included in the model. However, no significant differences were found when comparing current and past suicide attempters with the non-suicidal patients.

**Conclusions:**

Despite the small number of cases in the samples, our results confirmed the association of certain immunological parameters (NLR, MLR) and acute suicidal behaviour. This relationship was found to be independent of depression and its severity. Our data suggest that, unlike the NLR and MLR parameters, the higher CRP value may not be related to acute suicide attempt, but rather to suicidal vulnerability, as a trait-marker. Markers of chronic systemic inflammation may help in the prediction of suicidal behaviour and in the development of new therapeutic options, however, further prospective studies are needed to identify the specific role of immunological factors in suicidal behaviour more precisely.

**Disclosure of Interest:**

None Declared

